# Feasible study of MRgFUS of early breast cancer - report of completion of BC006 in Japan

**DOI:** 10.1186/2050-5736-3-S1-P45

**Published:** 2015-06-30

**Authors:** Kiyoshi Namba, Hiroyuki Kawami, Megumi Nakajima, Kenji Moriyama

**Affiliations:** 1 Hokuto Hospital, Obihiro, Japan

## Background/introduction

Among applications of MRgFUS such as uterine fibroid, breast cancer (BC), metastatice bone tumor, prostate, and the brain, the breast is prsently the only organ where complete local control of breast cancer is aimed. Immediately after completion of excision study (BC003, 2002-2003), one-arm prospective follow-up study to evaluate MRgFUS followed by adjunct radiation therapy (BC004) has been running wiht no local and systemic recurrences among 67 cases at Breastopia Namba Hospital in Japan. We report completion of excision study (BC006), the goal of which is, similar to BC003, prospective, non-randomized, single-arm, multiinternational study, and is to develop data to evaluate the safety and effectiveness of the Exablate 2000™ (InSightec, Israel) in the ablation of breast cancer by histopathological evaluation of MRI-guidance and the effect of FUS.

A prospective randomized 2-arm study of MRgFUS+radiation therapy (RT) and breast conserving surgery + RT will start September, 2014.

## Methods

Total of 5 eligible patients agreed to join this study after IC, May, 2012 - Feb, 2014 at Hokuto Hospital, Obihiro, Japan, after IRB’s approval. All lesions were diagnosed as discrete invasive ductal carcinoma wiht a maximal size of <2cm by ultrasound-guided vacuum biopsy (Vacora™ 10 gauge, BARD, USA) and 3T MRI (Signa HDxt, 3T, 8 channel Breast Coil, GE, USA). All the patients underwent ablation with MRgFUS. The goal of MRgFUS ablation of breast cancer is to plan and ablate the entire tumor volume in a treatable and device accessible location (lesion planned for ablation that is a minimum distance from unintended structures: dermal under surface, nipple complex and/or ribs shouls be 1.0 cm as defined by procedure day MR contrast-enhanced imaging, and with margins of 5-10mm by MRgFUS. MR imaging with and without MR contrast was performed at 10-21 days post ablation in order to assess the effectiveness of MRI in identifying residual disease. Pathological analyses will be performed on all excised samples to assess the endpoints of this study.

## Results and conclusions

Median age was 64 ± 13 (51 - 81). All were invasive ductal carcinoma and luminal A. MRgFUS was all tolerated with a minimum of adverse effects. On pathological examination, Median necrosis of the targeted breast tumors was 97.5% ± 2.5(95 - 100%) of tumor volume. The Median % area of carcinoma within the treatment field was 98 ± 2% (96% - 100%). Retrospective analysis in two patients with residual tumor showed treatment was not delivered to the full recommended area, reaffirming the need for precise localization and the value of contrast-enhanced images for treatment planning. Adverse effect were mild except one patient who complained of moderately uncomfortable feeling in the ablated area of the breast in one patient only during the treatment.

MRgFUS of early breast cancer was suggested to be effective and safe treatment.

